# Diversity of Gene Expression in Hepatocellular Carcinoma Cells

**DOI:** 10.1016/j.gpb.2015.07.003

**Published:** 2016-01-11

**Authors:** Fan Zhang, Li Cui, Michael D. Kuo

**Affiliations:** 1 Department of Molecular and Medical Genetics, University of North Texas Health Science Center, Fort Worth, TX 76107, USA; 2 Department of Neurosciences, School of Medicine, University of California, San Diego, La Jolla, CA 92093-0949, USA; 3 Department of Radiological Sciences, University of California, Los Angeles, David Geffen School of Medicine, Los Angeles, CA 90095, USA

**Keywords:** Gene expression, Hepatocellular carcinoma cell, Diversity, Microarray, Next-generation sequencing

## Abstract

Understanding tumor **diversity** has been a long-lasting and challenging question for researchers in the field of cancer heterogeneity or tumor evolution. Studies have reported that compared to normal cells, there is a higher genetic **diversity** in tumor cells, while higher genetic **diversity** is associated with higher progression risks of tumor. We thus hypothesized that tumor **diversity** also holds true at the **gene expression** level. To test this hypothesis, we used *t*-test to compare the means of Simpson’s diversity index for **gene expression** (SDIG) between tumor and non-tumor samples. We found that the mean SDIG in tumor tissues is significantly higher than that in the non-tumor or normal tissues (*P* < 0.05) for most datasets. We also combined **microarrays** and **next-generation sequencing** data for validation. This cross-platform and cross-experimental validation greatly increased the reliability of our results.

## Introduction

Cancer is a group of diseases characterized by uncontrolled division of abnormal cells [1]. Normal cells grow and divide in a controlled way; however, gene mutations can confer abnormal cells that no longer divide and reproduce in an orderly manner. Mutations in abnormal cells could activate oncogenes and inhibit tumor suppressors, thereby disrupting the normal balance between the two opposing processes of cell birth and cell death [2]. Most normal cells in tissues undergo programed cell death, called apoptosis, if detached from their neighboring cells. However, the self-destruct processes in cancer cells do not work; therefore, cancer cells are able to spread to other sites in the body (so-called metastasis) [3]. Cancer cells face selective pressure from their surrounding microenvironment that favors the survival of the fittest, which would be linked to the metastatic potential [4], [5].

Although human evolution and cancer progression are not identical, if cancer is considered in terms of evolution on a small time and spatial scale, a tumor cell population can be regarded as constantly evolving under natural selection [6]. For example, human evolution is driven by mutations in a gamete [7], while cancer is caused by mutations in the DNA of somatic cells; human evolution is a process of billions of years, while cancer progression occurs within the timescale of a human lifetime [8]. Maley et al. reported an evolutionary approach to characterize the diversity within a cell population in Barrett’s esophagus lesions [6]. They found that the increased diversity of tumor cells is strikingly correlated with the progression from normal cell to cancer [9]. On the one hand, tumor progression can be viewed as a sequential selection for fitter or dominant clones; on the other hand, tumors with greater genetic clonal diversity have a high probability of generating mutant cells, driving the transformation from the non-tumor to the tumor state [9], [10]. Cancer evolution is a reiterative process, which consists of clonal expansion, genetic diversification, and clonal selection, in the adaptive landscapes of tissue ecosystems [11].

Biodiversity is defined as the “variation of life at all levels of biological organization” [12], which not only involves the number of species, but also the number of individuals within each species. Diversity can be measured by Simpson’s diversity index (SDI), which takes into account the number of species and the abundance of each species. In this study, we applied the SDI to estimate the gene expression diversity in hepatocellular carcinoma (HCC) cells and its adjacent normal tissues.

HCC, the main type of primary liver cancer, is the most common cancer in some parts of the world [13] with rising incidence in the United States. The American Cancer Society estimated that about 35,660 people in the U.S. would be diagnosed with HCC, and about 24,550 people would die of the cancer in 2015 [14].

In this study, we first defined the proportion of positive sample (PPS) and the proportion of positive pair number (PPPN) of Simpson’s diversity index for gene expression (SDIG), and then a two-sample, one-sided *t*-test was performed to find out whether there was a significant difference in the mean SDIG between non-tumor and tumor tissues. We also combined microarray and the next-generation sequencing (NGS) data, which allows compensation, and cross validation of results obtained. Generally, microarrays are considered easier to use with less labor-intensive and less complicated sample preparation processes than those in NGS, whereas RNA-Seq technology offers better gene/transcript coverage. “In reality the two technologies couldn’t be more complementary”, as commented by Scott Peterson at J. Craig Venter Institute. The low cost, short turn-around time, exceptional quantitative accuracy, and ease of data generation all make the microarray the clear choice for gene expression study [15], to complement NGS studies.

## Materials and methods

### Data sources

We obtained six datasets by searching the Gene Expression Omnibus (GEO) [16] in 2015. These include (1) GSE5093 consisting of 20 normal and 20 tumor samples [17], (2) GSE3500 consisting of 76 non-tumor samples and 105 primary liver tumor samples [18], (3) GSE4024 consisting of 98 normal and 98 tumor samples [19], (4) GSE1898 consisting of 182 normal and 182 tumor samples [20], (5) GSE65484 consisting of 14 HCC patients and 14 paired adjacent normal samples [21], and (6) GSE65485 consisting of 50 HCC patients and 5 normal samples [21] (Table 1).

### Data processing

We first extracted gene expression values from the six datasets. In datasets GSE5093, GSE4024, and GSE1898, HCC and non-tumor sample were paired as channel 1 or channel 2. Mean intensity of each channel was calculated as shown below.

(1) ch1d_mean=ch1i_mean-ch1b_median

(2) ch2d_mean=ch2i_mean-ch2b_median

where, ch1i_mean and ch2i_mean are the uncorrected mean pixel intensity for channel 1 and channel 2, respectively; ch1b_median and ch2b_median are the median background pixel intensity for channel 1 and channel 2, respectively.

In the dataset GSE3500, the ratio of the intensity value in each sample to that in the reference sample was taken as its gene expression value. For example, if channel 1 is reference and channel 2 is sample (non-tumor or HCC), the gene expression value is ch2d_mean/ch1d_mean.

Next, if the signal quality was good, the flag value was set to 0, whereas flag values were set as −100 for poor signals and −50 for no signals. All non-flagged (flag value = 0) array elements with fluorescence intensities in each channel 1.5 times greater than the local background were considered well-measured, and all flagged (flag value = −50 or −100) array elements were removed.

Lastly, to eliminate noise and possible artifacts, we removed the genes for which measurements did not contain at least two replicates across the dataset (Table 1). Then, imputation was performed to eliminate the imbalance for the large ranges of the replicates.

The processed data were downloaded directly for NGS and aCGH analyses. We inversely transformed the log-transformed values in GSE65484 and used the fragments per kilobase million (FPKM) values in GSE65485 as gene expression values.

### Gene expression diversity index

Biodiversity indices represent the commonness and rarity of species in a community. The ability to measure diversity in this way enables biologists to understand the community structure.

SDI [22] is defined as

(3) $$D=1-\overset{S}{\underset{i=1}{\sum }}p_{i}^{2}\text{,}$$

where *D* refers to the Simpson’s index of diversity, *S* is the total number of species, and *p_i_* represents the proportion of the *i*th species.

The *p_i_* can be calculated by

(4) $$p_{i}=\frac{n_{i}}{N}=\frac{n_{i}}{\sum_{1}^{S}n_{i}}\text{,}$$

where *n_i_* is the number of individuals in the *i*th species and *N* is the total number of individuals in all the species.

We adapted three indices of diversity from the SDI in ecology and evolutionary biology into our study: gene number (*S*), proportion of expression value of gene *i* in relative to the total expression value of all genes (*p_i_*), and SDI for gene expression (*D*).

SDIG (*D*) is a simple mathematical measurement that characterizes gene expression diversity in a sample. *p_i_* is calculated by the following equation

(5) $$p_{i}=\frac{g_{i}}{\sum_{1}^{S}g_{i}}\text{,}$$

where *g_i_* is the expression value of the *i*th gene.

The squared proportions for all the genes are summed, subtracted from 1, and then SDIG (*D*) is calculated using equation (3). The index value ranges from 0 to 1; the greater the value is, the greater the gene expression diversity of sample is.

Let *DT_j_* be the SDIG of the *j*th sample in tumor tissue (*j* = 1,2,…*N*) and *DN_k_* be the SDIG of the *i*th sample in non-tumor or normal tissue (*k* = 1,2,…*M*). The total number of pairs (*DT_j_*, *DN_k_*) is *M* × *N*.

We define the positive pair of SDIG as those pairs of which SDIG in tumor tissue is greater than that in non-tumor or normal tissue.

The PPPN of SDIG relative to the total number of pair (*DT_j_*, *DN_k_*) is calculated by

(6) $$T=\frac{Q}{M\times N}\text{,}$$

where *Q* is the positive pair number of SDIG.

### Statistical analysis

A two-sample, one-sided *t*-test was performed to determine whether the mean SDIG in tumor tissues is greater than that in non-tumor or normal tissues. *P* values were determined by Welch’s *t*-test and differences are considered significant with *P* < 0.05.

## Results and discussion

The main purpose of this study was to test the hypothesis that the mean SDIG in HCC tumor tissues is higher than that in non-tumor or normal tissues. To do this, we first searched the GEO, the online resource for gene expression data, using the keyword “HCC” or “primary liver cancer”, and retrieved six HCC-related datasets. These include GSE5093 [17], GSE3500 [18], GSE4024 [19], GSE1898 [20], GSE65484 [21], and GSE65485 [21]. Then, we defined the SDIG, PPS, and PPPN. Lastly, we used *t*-test to compare the mean SDIG between HCC tumor and non-tumor or normal tissues.

We defined PPS for these datasets, in which the gene expression diversity of primary liver tumor channel is greater than that of the non-tumor channel. No PPS was calculated for GSE3500 and GSE65485 datasets, since non-tumor samples were not paired with HCC samples. Alternatively, we calculated the PPPN by SDIG and employed *t*-test to compare gene expression diversity between the non-tumor and HCC samples.

**Figure 1:**
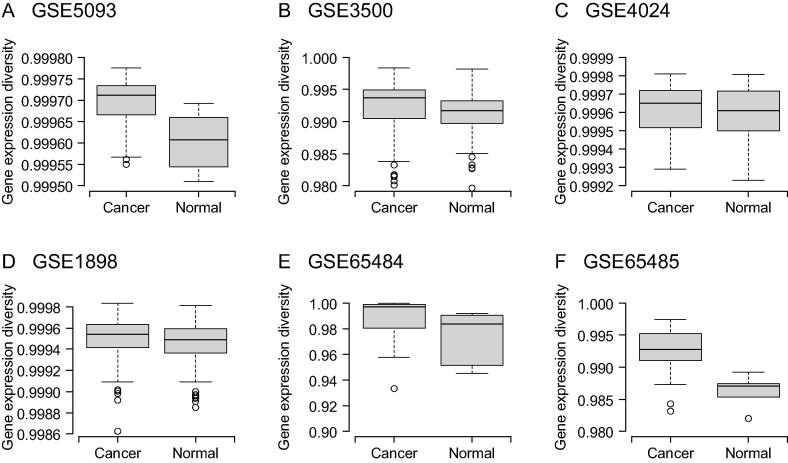
**Gene expression diversity of HCC tumor and non-tumor samples in the six datasets examined** Boxplot of gene expression diversity for GSE5093 (**A**), GSE3500 (**B**), GSE4024 (**C**), GSE1898 (**D**), GSE65484 (**E**), and GSE65485 (**F**), respectively. The median expression diversity is indicated with horizontal line. The boxplot shows minimum, first quartile, median, third quartile, and maximum from bottom to top. The gene expression diversity was calculated using equations (3), (4), (5).

Gene expression diversity in both non-tumor (normal) and HCC tumor tissues for the six datasets is shown in Figure 1. Median SDIG in tumor tissue is greater than that in non-tumor or normal tissues for GSE5093, GSE3500, GSE1898, GSE65484, and GSE65485. Table 2 shows that all the PPPN of SDIG are greater than 50%. The mean *T* (PPPN) value is 72%, which is nearly 3 times as much as the proportion of the negative pair number (PNPN) (28%). PNPN represents the proportion of the pairs in which SDIG of tumor tissue is not greater than that in non-tumor or normal tissue, relative to the total number of pairs. This result indicates that for a majority of sample pairs, the gene expression diversity in HCC samples is greater than that in non-tumor samples.

We lastly used the *t*-test to determine whether the mean of SDIG in tumor tissue is greater than that in the non-tumor or normal tissue. It was shown that except for the GSE4024, gene expression diversities in tumor tissues were significantly higher than those in non-tumor or normal tissues in all the other five datasets (*P* < 0.05). In particular, highly significant gene expression diversities were observed for GSE5093, GSE1898, and GSE65485 (*P* < 0.01). Datasets GSE4024 and GSE1898 came from the same lab with same RNA preparation and microarray procedure [19], [20], but contained different numbers of samples. There were 196 and 364 samples for GSE4024 and GSE1898, respectively. We speculate that the smaller sample size in GSE4024 could partially explain the different observations between these two datasets, since the confidence in the hypothesis increases when the sample size increases.

According to the aforementioned PPPN and *t*-test analyses for gene expression diversity in both non-tumor and HCC tumor tissues, we found greater gene expression diversity in HCC tumor samples than the non-tumor samples. This observation is consistent with previous finding [23]. Using sequencing-based gene expression profiles (SAGE-seq), Wu et al. tested the gene expression diversity in breast cancer and found that breast cancer samples have higher diversity than that from normal samples [23]. Different from their study on breast cancer samples, our study on the same assumption is exclusively focused on HCC. Secondly, only 14 samples were included in their study, while we tried to collect as many HCC samples as possible from publically available repositories such as GEO and The Cancer Genome Atlas (TCGA). Since the 423 liver cancer samples in TCGA provided only level 3 data, which contain normalized read counts instead of gene expression value, we didn’t include any TCGA datasets to our study. As a result, we collected six GEO datasets (totally 864 samples) for HCC gene expression data from HCC and adjacent non-tumor samples. Thirdly, their conclusion was based on Wilcoxon rank-sum test’s *P* value of 0.07284, which is in the borderline of significance due to limited number of samples, while in our study, validation based on the cross-platform and cross-experiment largely increases reliability for our study and we chose more stringent significances value of 0.05 and 0.01 as cutoff.

Testing the gene expression diversity in HCC is a first-time study up to now. Further experiments on this may lead to better understanding of the relationship between increased gene expression diversity and the processes involved in cancer progression from non-tumor to tumor in HCC. There is a high probability that the diversely expressed tumor gene causes uncontrolled gene pathway regulation [24], which may drive the transformation from non-tumor to HCC.

Moreover, “the more evenly distributed gene expression, the higher its diversity” [25]. Gene expression in normal liver cells may be distributed randomly [26]. However, it may become more evenly distributed in HCC; that is, functionally important genes may be expressed as equally as the ubiquitous genes in the progression from non-tumor to HCC. A more evenly distributed expression of oncogenes and tumor suppressors may trigger cancer by disturbing the normal balance between cell mitosis and apoptosis [27]. Measurement of gene expression diversity may assist in finding biomarkers for cancer risk and progression from non-tumor to HCC, through the accumulation of evenly-expressed genes.

One limitation of the current study is that gene ontology (GO) and pathway analyses were not performed. In the future, we will look into the pathways, gene sets, or modules to understand if higher gene expression diversity is concentrated in certain cellular pathways or more conserved in certain pathways.

## Conclusion

HCC is the most common cancer and the leading cause of death in some parts of the world. In this study, we applied the diversity index used in the measurement of biodiversity to gene expression of non-tumor and HCC samples, for six datasets obtained from GEO. We used PPS, PPPN of SDIG, and a two-sample, one-sided *t*-test to prove that gene expression diversity in HCC samples is higher than that in non-tumor samples.

## Authors’ contributions

FZ and MK conceived the initial work and designed the method. FZ developed the gene expression diversity index method and performed all the computational analyses and drafted the manuscript. LC performed web search and downloaded all HCC-related data. All authors were involved in the manuscript editing and revision, read and approved the final manuscript.

## Competing interests

The authors have declared no competing interests.

## Figures and Tables

**Table 1:** Main characteristics of datasets examined in the current study

**Dataset**	**Platform**	**No. of tissue samples**			**Data removed (%)**	**No. of replicates average (range)**	**Note**	**Ref.**
**Total**	**Tumor**	**Normal**
GSE5093	Microarray	40	20	20	1.24	38 (3–40)	Tumor samples and the corresponding non-cancerous adjacent hepatic tissues came from two HCC patient groups, *i.e.*, MIM with primary HCC and venous metastasis and MAM with HCC but no detectable metastasis	[17]

GSE3500	Microarray	181	105	76	9.86	172 (3–180)	The dataset included expression data in more than 200 samples. 105 tumor samples from 82 HCC patients with primary HCC and 76 non-tumor samples from 72 controls were analyzed in this study for genes that were shared by all the 181 samples	[18]

GSE4024	Microarray	196	98	98	9.83	192 (3–196)	RNA from 19 normal liver samples was pooled as reference for all microarray experiments. At least two hybridizations were carried out to obtain gene expression profile data for each of the 49 HCC tissues	[19]

GSE1898	Microarray	364	182	182	8.31	360 (3–364)	RNA from 18 normal liver samples was pooled as reference for gene expression profiles from 91 human HCC tissues. Two hybridizations were performed for each of the 91 HCC tissues	[20]

GSE65484	aCGH	28	14	14	0		The dataset included 50 HCC patients and 14 paired adjacent tissues We only used the 14 pairs of HCC and normal tissues for our study	[21]

GSE65485	NGS	55	50	5	0		Whole transcriptome sequencing profiling was performed for 50 HCC samples and 5 adjacent normal samples	[21]

*Note:* aCGH, array-based comparative genomic hybridization; NGS, next-generation sequencing; MIM, missing in metastasis; MAM, metachronous adrenal metastasis.

**Table 2:** Gene expression diversity in HCC and normal samples of datasets examined in the current study

**Dataset**	**Data structure**	**PPS (%)**	***Q***	***T* (%)**	**Mean gene expression diversity**		***P* value**
**Normal**	**HCC**
GSE5093	Paired	100	343	86	0.9996	0.9997	3.756E−05^∗^
GSE3500	Non-paired	NA	5150	65	0.9894	0.9918	0.0178^∗^
GSE4024	Paired	61.2	5283	55	0.9996	0.9996	0.1034
GSE1898	Paired	67.2	19,543	59	0.9995	0.9995	0.0032^∗^
GSE65484	Paired	85.7	146	74.5	0.9420	0.9861	0.0495^∗^
GSE65485	Non-paired	NA	238	95.2	0.9862	0.9927	0.0018^∗^
Mean				72			

*Note:* GSE3500 and GSE65485 are not paired and PPS can’t be calculated. *Q* indicates the positive pair number of SDIG, and *T* indicates the proportion of positive pair number (PPPN) of SDIG relative to the total number of tumor and normal SDIG pairs. HCC, hepatocellular carcinoma; PPS, proportion of positive sample; NA, not available; SDIG, Simpson’s diversity index for gene expression. *t*-test was performed for statistical analysis and differences between HCC and normal samples are considered significant with *P *< 0.05 (^∗^).
